# Imaging and procedural features of staged bilateral magnetic resonance-guided focused ultrasound thalamotomy for essential tremor

**DOI:** 10.3389/fradi.2026.1779005

**Published:** 2026-03-27

**Authors:** Fabio Paio, Antonio Ambrosin, Giorgia Bulgarelli, Micaela Tagliamonte, Chiara Zucchella, Elisa Mantovani, Tommaso Bovi, Michele Longhi, Antonio Nicolato, Emanuele Zivelonghi, Luisa Altabella, Carlo Cavedon, Francesco Sala, Benedetto Petralia, Bruno Bonetti, Michele Tinazzi, Stefano Tamburin, Giuseppe K. Ricciardi

**Affiliations:** 1 Neurology Section, Department of Neurosciences, Biomedicine, and Movement Sciences, University of Verona, Verona, Italy; 2 Neurology Unit, Department of Neurosciences, Azienda Ospedaliera Universitaria Integrata, Verona, Italy; 3 Neuroradiology Unit, Department of Pathology and Diagnostics, Azienda Ospedaliera Universitaria Integrata, Verona, Italy; 4 Stereotactic Neurosurgery and Radiosurgery Unit, Department of Neurosciences, Azienda Ospedaliera Universitaria Integrata, Verona, Italy; 5 Medical Physics Unit, Department of Pathology and Diagnostics, Azienda Ospedaliera Universitaria Integrata, Verona, Italy; 6 Neurosurgery Section, Department of Neurosciences, Biomedicine, and Movement Sciences, University of Verona, Verona, Italy; 7 Neurosurgery Unit, Department of Neurosciences, Azienda Ospedaliera Universitaria Integrata, Verona, Italy

**Keywords:** bilateral thalamotomy, essential tremor, magnetic resonance-guided focused ultrasound, MRgFUS, volume lesion

## Abstract

**Background:**

Magnetic Resonance–guided Focused Ultrasound (MRgFUS) thalamotomy is an established incisionless treatment for medication-refractory essential tremor (ET). While staged-bilateral MRgFUS thalamotomy has recently gained clinical acceptance, detailed radiological and procedural comparisons between first- and second-side interventions remain limited. This study aimed to provide a comprehensive imaging-based and procedural characterisation of staged bilateral MRgFUS thalamotomy, with a specific focus on stereotactic targeting, sonication parameters, and lesion morphology.

**Materials and methods:**

In this retrospective-prospective, single-centre observational study, consecutive patients with ET undergoing staged bilateral MRgFUS thalamotomy were included. Procedural metrics, stereotactic targeting coordinates, and sonication parameters were compared between FUS1 and FUS2 procedures. MRI analyses were performed using a standardised protocol including morphological pre-treatment 3D T1-weighted imaging and post-treatment 3D T2-weighted imaging acquired within 24 h and at approximately 1 month to document the lesion. Treatment-related lesions were segmented using a semi-automated approach, with volumetric measurements independently obtained by two blinded raters; inter-rater agreement was assessed using intraclass correlation coefficients. Adverse events (AEs) were recorded as secondary outcomes. A systematic review of the literature on treatment strategy of staged bilateral MRgFUS thalamotomy was conducted.

**Results:**

Fifteen patients underwent staged bilateral MRgFUS thalamotomy. Most anatomical, procedural, and sonication-related parameters were comparable between FUS1 and FUS2. Final stereotactic targeting during FUS2 showed a small but consistent anterior and dorsal shift relative to FUS1. Lesion volumes measured at both 24 h and 1 month after the procedure did not differ significantly between FUS1 and FUS2, and inter-rater agreement for lesion volumetry was excellent across time points (ICC > 0.91). AEs after FUS2 were predominantly mild and transient. We found no significant differences in lesion volume or inter-side targeting displacement between patients with and without gait disturbances, the most common AE, persisting at 1 month after FUS2. Single-patient imaging analyses suggested heterogeneous spatial lesion configurations in patients with AEs persistent 6–12 months after FUS2.

**Conclusion:**

In this single-centre cohort, staged bilateral MRgFUS thalamotomy showed high procedural and radiological consistency between FUS1 and FUS2. MRI-based volumetric analyses show consistent lesion morphology across hemispheres, with small, safety-oriented refinements in second-side targeting, in line with the literature.

## Introduction

Magnetic Resonance–guided Focused Ultrasound (MRgFUS) thalamotomy is an incisionless treatment for medication-refractory essential tremor (ET), enabling stereotactic lesioning of thalamic targets under real-time MRI guidance and thermal monitoring. From a procedural and imaging standpoint, MRgFUS offers unique advantages compared with historical ablative techniques, i.e., submillimetric precision, intraoperative thermal monitoring, and post-procedural MRI-based lesion characterisation.

Over the past decade, randomised trials and prospective studies have established the clinical efficacy and durability of unilateral MRgFUS thalamotomy, leading to its widespread adoption (1, 2). Historically, however, bilateral thalamotomy performed using traditional radiofrequency lesioning was associated with a high risk of adverse events (AEs), including dysarthria and gait instability, which limited its clinical applicability (3). The advent of MRgFUS has renewed the feasibility of bilateral thalamotomy, with recent prospective trials and multicentre series supporting its feasibility and safety when procedures are staged, i.e., temporally separated by at least 6–9 months (4–6).

Despite evidence of clinical effectiveness and safety, radiological and procedural data on staged bilateral MRgFUS thalamotomy remain limited. Systematic comparisons between first- and second-side interventions regarding stereotactic targeting strategies, sonication characteristics, lesion size and spatial distribution are scarce. Moreover, the temporal evolution of treatment-related lesions and the reproducibility of volumetric measurements across staged procedures have not been comprehensively investigated in real-world cohorts.

Therefore, the present study was designed to provide a detailed procedural and imaging-based characterisation of staged bilateral MRgFUS thalamotomy in patients with ET. We specifically compared stereotactic coordinates, sonication parameters, and lesion volumes between first- and second-side procedures, with particular emphasis on lesion morphology and spatial consistency on serial MRI. AEs were analysed as secondary outcomes. Efficacy on tremor was not reported as a secondary outcome because the efficacy of staged-bilateral MRgFUS was the topic of a separate study (7). Further, a systematic review of the literature of procedural and radiological evidence on bilateral MRgFUS thalamotomy was performed.

## Methods

### Study design

This was a retrospective-prospective, single-centre observational study conducted at the University Hospital of Verona, including consecutive patients with ET who underwent staged bilateral MRgFUS thalamotomy. All patients received a first-side MRgFUS thalamotomy (FUS1) between July 2018 and May 2024 and subsequently underwent a contralateral second-side MRgFUS thalamotomy (FUS2) between November 2023 and March 2025. Access to FUS occurred within routine clinical practice pathways. Patients were evaluated for contralateral treatment either during scheduled post-FUS1 follow-up or following referral back to the centre. The interval between FUS1 and FUS2 varied across patients and, in some cases, extended over several months, reflecting regulatory approval timelines and real-world access.

Clinical and radiological data were collected retrospectively for the first procedure (FUS1) and prospectively for the second procedure (FUS2). For radiological analyses, pre-treatment MRI, early post-treatment MRI (within 24 h), and late post-treatment MRI (approximately 1 month) were analysed for both FUS1 and FUS2. Regarding clinical data, the analyses were focused on FUS2, for which baseline and follow-up assessments at 1, 6, and 12 months were collected prospectively. Although clinical efficacy measures, including tremor severity assessed by the Clinical Rating Scale for Tremor (8), were routinely collected as part of standard clinical care, they were not analysed in the present study, which was specifically designed to address radiological and procedural aspects of staged bilateral MRgFUS thalamotomy. Clinical outcomes were therefore limited to safety analyses. AEs were evaluated as secondary outcomes and classified by domain and severity according to the Common Terminology Criteria for Adverse Events, version 5 (CTCAE v5) (9) as detailed in Supplementary Table S1. In addition, axial function was explored using selected items (i.e., items 1–4) of the Scale for the Assessment and Rating of Ataxia (10), which were routinely collected before FUS2 and at each post-procedural follow-up visit.

### MRgFUS procedure

All procedures were performed using the Exablate Neuro 4000 system (Insightec, Haifa, Israel), integrated with a 3-Tesla MRI scanner (Signa Architect; GE Healthcare, Milwaukee, WI, USA). Premedication with steroids, head shaving, stereotactic frame placement, coupling with the ultrasound helmet, and preparation of the circulating water system followed standardised protocols previously adopted at our centre (11, 12). For FUS2, the initial target was defined as the mirror image of the first-side lesion relative to the interhemispheric midline, using the stereotactic coordinates of FUS1 as reference. This symmetric target served as a starting point and was subsequently refined intraoperatively based on MR thermometry and clinical testing following low-power sonications. As in FUS1, sonications were delivered in a stepwise fashion, with gradual increases in acoustic power and duration to achieve therapeutic temperatures, while repeatedly monitoring tremor suppression and the occurrence of transient neurological signs. In most patients, the definitive target for FUS2 was ultimately adjusted slightly dorsally relative to the initial ablation point, in line with our centre's experience, to minimise the risk of adverse effects associated with more ventral lesioning, while refinement along the X- and Y-axes was driven by intraoperative clinical response. The decision to stop MRgFUS sonications was made when a clear tremor benefit was observed.

For each procedure (i.e., FUS1 and FUS2), the duration of the intervention (from first to last sonication), skull density ratio (SDR) derived from pre-procedural CT, and stereotactic metrics, including the anterior–posterior commissural distance and an index of third ventricle size on preoperative MRI, were recorded. Procedural parameters included the total number of sonications, average sonication duration, mean delivered energy, mean acoustic power, and mean and maximum temperatures ≥ 55 °C.

### MRI acquisition and post-procedural follow-up

All patients underwent a standardised MRI protocol on a 3 Tesla scanner. For each treated side (FUS1 and FUS2), three volumetric 3D MRI sequences were selected for analysis: a pre-treatment morphological T1-weighted BRAVO sequence (T1 pre), and a 3D T2-weighted CUBE sequence acquired within 24 h after the procedure (T2 early) and approximately one month after treatment (T2 late), to characterise treatment-related lesions and perform all volumetric and spatial analyses. Detailed MRI acquisition protocols are provided as Supplementary Material.

#### Image preprocessing

Preprocessing was performed using 3D Slicer (13) (version 5.8.1). The selected MRI sequences were first imported in DICOM format and converted into Neuroimaging Informatics Technology Initiative (NIfTI) format. An automated skull stripping step was then applied using the HD Brain Extraction tool to remove non-brain tissues and obtain brain-only images suitable for subsequent registration and segmentation. For each patient, T2 early and T2 late images were rigidly co-registered to the corresponding T1 pre image using the “General Registration” module from the Advanced Normalization Tools (ANTs) library (14). The registration was performed with the “QuickSyN” setting for the stages and using the “images geometric center” as the initial moving transform, as specified in the original pipeline.

#### Thalamic and lesion segmentation

**Figure 1:**
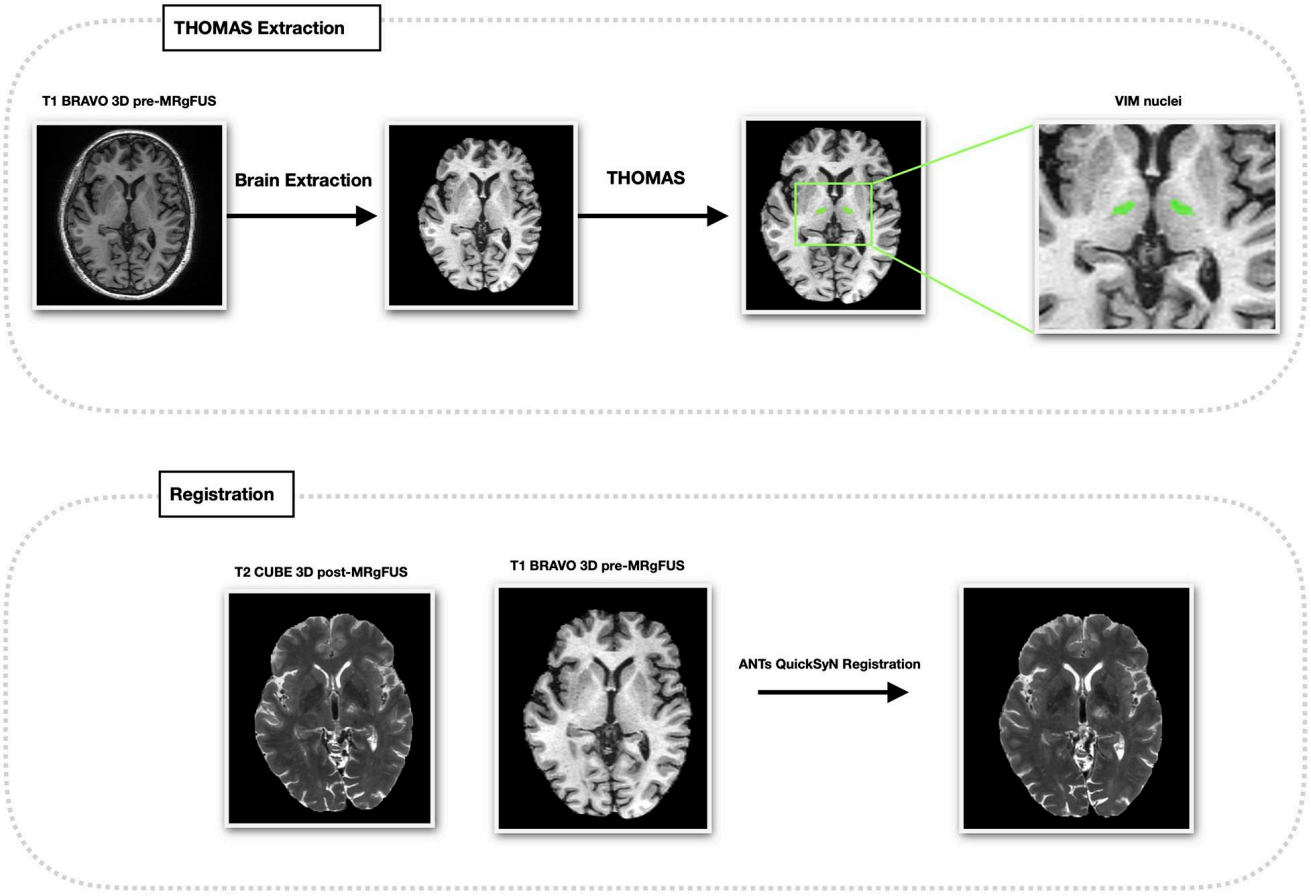
Thalamic segmentation, target definition, and image registration. For each patient pre-treatment T1-weighted 3D BRAVO images were processed with automated brain extraction followed by thalamic nuclei segmentation using the THalamus Optimized Multi-Atlas Segmentation software, integrated with HIstogram-based Polynomial Synthesis (HIPS-THOMAS). The ventral lateral posterior nucleus, ventral portion (VLPv), corresponding to the Vim nucleus according to the Hirai and Jones nomenclature, is shown and highlighted. Post-treatment 3D T2-weighted CUBE images acquired within 24 h (T2 early) and at approximately one month (T2 late) after FUS1 and FUS2 were rigidly coregistered to the corresponding pre-treatment T1-weighted image using the “General Registration” module from the Advanced Normalization Tools (ANTs) library with the QuickSyN registration setting.

**Figure 2:**
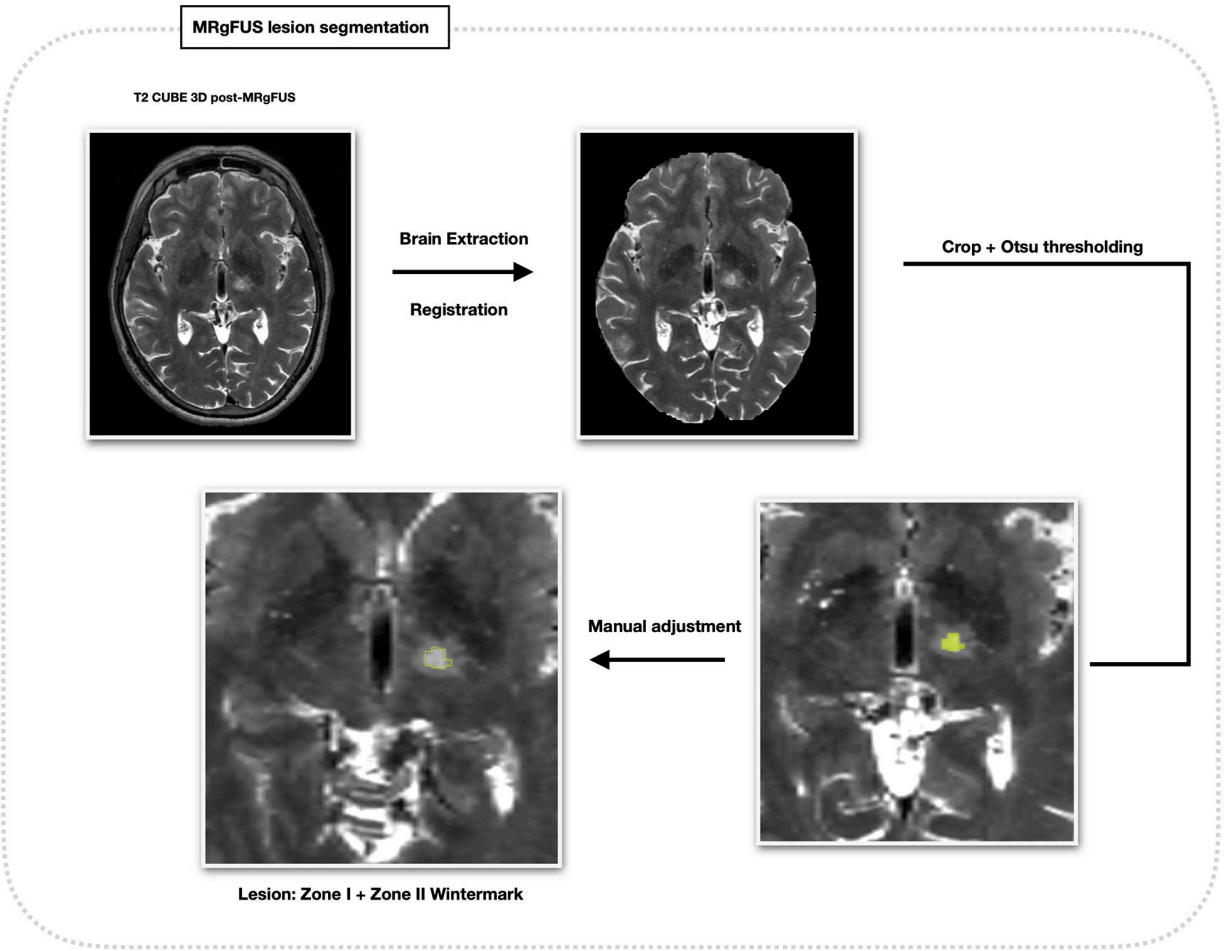
MRgFUS lesion segmentation workflow. Treatment-related lesions were segmented on the co-registered 3D T2-weighted CUBE images by combining Wintermark zones I and II, corresponding to the necrotic core and the surrounding cytotoxic oedema. Lesion segmentation was performed on images acquired within 24 h (T2 early) and at approximately one month (T2 late) after FUS1 and FUS2. A cubic region of interest was manually defined around each lesion, followed by threshold-based segmentation using Otsu's method and manual refinement when required.

Thalamic nuclei were segmented on T1 pre images using the THalamus Optimized Multi-Atlas Segmentation software, integrated with HIstogram-based Polynomial Synthesis (HIPS-THOMAS) (15, 16). The nuclei of interest were the ventral lateral posterior nucleus, ventral portion (VLPv), corresponding to the Vim nucleus according to the Hirai and Jones nomenclature (17) (Figure 1). On the coregistered T2 early and T2 late images, treatment-related lesions were segmented by considering the combined extent of zones I and II, corresponding to the central necrotic core and the surrounding cytotoxic oedema (18). In detail, a cubic region of interest (ROI) was manually drawn around each lesion, and threshold-based segmentation using Otsu's method was applied within the ROI. When necessary, thresholds were manually adjusted to avoid obvious over- or under-segmentation (Figure 2). Final lesion masks were visually inspected and refined using manual correction tools available in 3D Slicer.

#### Volumetric measurements

Volumetric measurements were obtained using dedicated tools in 3D Slicer applied to the segmented thalamic nuclei and lesions. For each treated side and time point, lesion volumes were calculated on T2 early and T2 late images, whereas the volume of the Vim (VLPv) nucleus was calculated on T1 pre images.

Lesion segmentation and volume measurements were performed using a semi-automated approach by two independent and blinded raters (i.e., a senior resident and an experienced neuroradiologist). Inter-rater agreement was assessed using the intraclass correlation coefficient (ICC), with a two-way random-effects model for absolute agreement (ICC[2,1]). Agreement was further explored using Bland–Altman analysis to estimate mean bias and limits of agreement (19).

### Statistical analysis

Statistical analyses were performed using R software (version 4.5.0; R Foundation for Statistical Computing, Vienna, Austria) (20). For within-subject comparisons between FUS1 and FUS2, the distribution of within-subject differences was evaluated with Shapiro–Wilk test: paired *t*-tests were used when differences were approximately normally distributed, whereas the Wilcoxon signed-rank test was applied otherwise and effect sizes were estimated using Wilcoxon rank-biserial correlation. For consistency across procedural and imaging parameters, continuous variables in paired analyses are reported as mean ± standard deviation (SD), regardless of the statistical test applied.

Between-group comparisons were performed using the Wilcoxon–Mann–Whitney test, given the small sample size and the non-normal distribution of most variables; results were reported as median and interquartile range (Q1–Q3).

All tests were two-sided, and a significance level of 0.05 was adopted.

### Anatomical correlates of adverse events

An exploratory subgroup analysis was performed to explore potential radiological correlates of gait disturbances as AEs after FUS. Patients were stratified according to the presence or absence of gait disturbances persisting at 1-month follow-up after FUS2, and lesion volumes measured in T2 early and T2 late after FUS2 were compared between the two groups. For each patient, the Euclidean distance between the final stereotactic coordinates of the centre of lesions after FUS1 and FUS2 was calculated in a common AC–PC–based reference space. The distance was computed as the square root of the sum of squared differences along the three orthogonal stereotactic axes (x, y, z), according to the following formula:

$$d=\sqrt{(x_{FUS2}-x_{FUS1}{)}^{2}+{\left(y_{FUS2}-y_{FUS1}\right)}^{2}+{\left(z_{FUS2}-z_{FUS1}\right)}^{2}}$$

Where x, y, and z represent the final stereotactic coordinates of the targeting point for each procedure. This metric was adopted as an exploratory and indirect surrogate of inter-side spatial targeting symmetry relative to the AC–PC reference space, with higher values indicating greater asymmetry between first- and second-side targeting, and values approaching zero reflecting increasingly symmetric spatial alignment. In addition, between-group descriptive and exploratory analyses were performed to assess whether patients with and without gait disturbances differed for clinical, demographic, or procedural features.

An exploratory single-patient analysis was conducted in patients presenting with persistent AEs of any type at the last available follow-up. For each of these patients, lesion volumes on early and late T2-weighted MRI after FUS2 were reported and descriptively compared with the corresponding group mean values. Spatial characteristics were further explored by calculating the Euclidean distance between the final stereotactic coordinates of FUS1 and FUS2 for each individual patient. Lesion location was visually represented only on late (1-month) T2-weighted MRI by overlaying lesion masks onto the corresponding thalamic segmentation, to qualitatively assess lesion position relative to the Vim nucleus and adjacent structures.

### Systematic review of the literature

A systematic review of studies assessing staged bilateral MRgFUS thalamotomy in patients with medically refractory ET was conducted in accordance with the PRISMA 2020 guidelines. PubMed, Web of Science Core Collection, and the Cochrane Library were searched up to 15 December 2025 using the following search string: (“magnetic resonance imag* guided focus* ultrasound” OR “magnetic resonance guided focus* ultrasound” OR MRgFUS OR “high intensity focus* ultrasound” OR HIFU) AND (bilateral OR staged) AND (tremor). All clinical studies reporting bilateral MRgFUS thalamotomy for essential tremor were considered eligible. Review articles and non-English publications were excluded. The primary focus of the qualitative synthesis was on targeting strategies and anatomical approaches.

## Results

### Study population

Fifteen right-handed patients (60% men) underwent staged bilateral MRgFUS thalamotomy. The mean age at surgery was 71.8 ± 8.6 years (range, 51–84) for FUS1 and 74.1 ± 8.9 years (range, 52–89) for FUS2. The mean interval between FUS1 and FUS2 was 28.9 ± 22.5 months (range, 10–69).

Baseline clinical and radiological data from FUS1 were unavailable for one patient (pt. n°6), as the FUS1 had been performed at another Centre. In another patient (pt. n°10), radiological data for FUS1 could not be retrieved because they were not available in the Institutional archive.

At the 12-month time point, clinical data were available for 10 of the 15 patients. For the remaining five patients, follow-up data were available up to 6 months only, as they had not yet reached the 12-month visit according to the study calendar; no patients were lost to follow-up.

Clinical tremor scores, routinely collected as part of standard clinical care, are provided in Supplementary Table S2.

### Main results

Most anatomical, procedural, and targeting-related parameters were comparables between FUS1 and FUS2 (Table 1). No significant differences were observed in SDR, anterior–posterior commissural distance, or third ventricle diameter between the two interventions.

**Table 1:** Main procedural and radiological data.

Variables	FUS1	FUS2	*p*-value
SDR	0.53 ± 0.07	0.53 ± 0.07	*p* = 0.61^a^
ACPC length (mm)	26.2 ± 2.5	26.0 ± 2.3	*p* = 0.81^a^
Third ventricle diameter (mm)	7.95 ± 2.4	7.97 ± 2.39	*p* = 0.54
Starting coordinates^b^ (mm)			
x	14.6 ± 0.7	14.6 ± 0.7	*p* = 1^a^
y	7.5 ± 0.5	7.8 ± 0.7	***p*** **=** **0.04**
z	1.4 ± 0.4	1.4 ± 0.3	*p* = 0.57^a^
Final coordinates^b^ (mm)			
x	14.7 ± 1.1	14.6 ± 0.8	*p* = 0.27
y	7.9 ± 1.0	8.4 ± 0.7	*p* = 0.06
z	1.6 ± 0.5	2.0 ± 0.4	***p*** **=** **0.006**^a^
Procedure duration (min)	66 ± 21	57 ± 25	*p* = 0.35
Total N of sonications	9.6 ± 3.3	8.2 ± 2.6	*p* = 0.09
N of sonications without alignment	6.2 ± 2.4	5.6 ± 2.0	*p* = 0.12
Parameters of sonications with T mean ≥ 55 °C^^^			
N of sonications	2.1 ± 0.8	1.9 ± 0.9	*p* = 0.66^a^
Sonication duration (sec)	17.5 ± 3.7	19.3 ± 7.7	*p* = 0.50^a^
Mean Energy (J)	13,692.9 ± 4,287.9	13,551.1 ± 6,511.2	*p* = 0.86
Mean Power (W)	779.7 ± 78.6	726.7 ± 132.8	*p* = 0.16
Mean T ( °C)	55.6 ± 2.1	54.7 ± 2.7	*p* = 0.11
Max T ( °C)	58.5 ± 2.5	58.5 ± 4.2	*p* = 0.92
Lesion volume^d^			
Within 24 h after the procedure	77.7 ± 20.0	79.3 ± 33.4	*p* = 0.80
At 1 month after the procedure	76.5 ± 38.7	88.0 ± 39.6	*p* = 0.40^a^

Data are presented as mean ± standard deviation (SD), for descriptive consistency. *P* values refer to paired comparisons between FUS1 and FUS2 and were derived using a paired t test when the distribution of within-subject differences was approximately normal; otherwise, the Wilcoxon signed-rank test was applied.

^a^ *p-*values derived using the Wilcoxon signed-rank test.

^b^ *Stereotactic coordinates* are expressed as follows: x, distance (mm) from the AC–PC line; y, distance (mm) from the posterior commissure; z, distance (mm) orthogonal to the AC–PC plane. Final stereotactic coordinates reflect the intraoperative target refinement based on real-time MRI thermometry and clinical testing.

^c^ When the target mean temperature of 55 °C could not be achieved, sonications with the highest delivered acoustic power, reaching a mean temperature of at least 50C, were included for descriptive analysis.

^d^ *Volume lesion values* derived from lesion segmentation represent the mean of measurements obtained independently by the two raters.

ACPC: anterior commissure-posterior commissure; min: minutes; mm: millimetres; N: number; J: Joule; SDR: skull density ratio, sec: seconds; W: Watt.

Statistically significant *p*-values are shown in bold.

With respect to stereotactic targeting, starting coordinates were largely overlapping between FUS1 and FUS2, apart from the y-coordinate, which was slightly but significantly more anterior in FUS2 (7.8 ± 0.7 mm vs. 7.5 ± 0.5 mm; *p* = 0.04), and similar to the final FUS1 y-coordinate. Final coordinates showed a tendency to more anterior (y: 8.4 ± 0.7 mm vs. 7.9 ± 1.0 mm; *p* = 0.06) and significantly more dorsal (z: 2.0 ± 0.4 mm vs. 1.6 ± 0.5 mm; *p* = 0.01) position in FUS2 compared with FUS1, while medio-lateral (x) coordinates did not differ significantly.

Procedure duration tended to be shorter during FUS2, although this difference did not reach statistical significance. Similarly, the total number of sonications and the number of sonications performed without target realignment were slightly but not significantly lower during FUS2. Sonication parameters associated with therapeutic temperatures (i.e., mean temperature ≥ 55 °C) were comparable between procedures, including the number of sonications, sonication duration, delivered energy, acoustic power, and achieved mean and maximum temperatures. However, in a limited subset of patients, a mean temperature ≥ 55 °C could not be reached during the procedure. Specifically, two patients did not reach this threshold during either FUS1 or FUS2, and four additional patients did not reach it during FUS2, despite having reached this threshold in FUS1. In all these cases, sonications were stopped because a clear and satisfactory clinical benefit had already been achieved during intraoperative testing, and further thermal escalation was judged unnecessary and potentially risky.

Lesion volumes measured in T2 early were comparable between FUS1 and FUS2 (77.7 ± 20.0 mm^3^ vs. 79.4 ± 33.4 mm^3^; *p* = 0.80). In T2 late, lesion volumes were similar between procedures (76.5 ± 38.7 mm^3^ vs. 88.0 ± 39.6 mm^3^; *p* = 0.40).

Inter-rater agreement for lesion volume measurements was consistently high across both procedures and imaging time points (Supplementary Table S3). ICC[2,1] indicated excellent reliability overall, ranging from 0.91 to 0.95 for T2 early and increasing to ≥0.99 for T2 late in both FUS1 and FUS2. Bland–Altman analysis demonstrated minimal mean bias between raters and narrower limits of agreement for T2 late compared to T2 early, indicating reduced measurement variability and improved consistency of lesion delineation over time.

### Adverse events

At the baseline evaluation prior to FUS2, none of the patients had persistent AEs related to FUS1. Overall, AEs after FUS2 were predominantly mild and transient (CTCAE grade 1–2). Most AEs occurred within the first month and progressively decreased over time, as shown in Table 2. Gait disturbance was the most frequent AE, occurring in up to 80% of patients within the first days after the procedure and reported in 53.3% (*n* = 8) of patients at 1 month. Less frequent AEs, mainly observed within the first month, included fatigue, steroid-related effects (tachycardia, mild hyperglycaemia, and oral candidiasis), and difficulty in word finding. At the last available follow-up, only a small number of mild AEs persisted, predominantly involving speech- and sensory-related symptoms (i.e., dysgeusia and finger hypoesthesia).

**Table 2:** Type, severity, and temporal evolution of adverse events after FUS2.

AEs	Gait disturbance			Dysarthria			Dysphagia			Dysgeusia			Dysesthesia			Muscle weakness		
FU	1	6	12	1	6	12	1	6	12	1	6	12	1	6	12	1	6	12
Pt. 1	0	0	0	0	0	0	0	0	0	0	0	0	0	0	0	0	0	0
Pt. 2	1	1	0	2	1	1	0	0	0	0	0	0	0	0	0	0	0	0
Pt. 3	1	0	0	0	0	0	0	0	0	0	0	0	1	1	1	0	0	0
Pt. 4	0	0	0	0	0	0	0	0	0	0	0	0	0	0	0	0	0	0
Pt. 5	1	0	0	0	0	0	0	0	0	0	0	0	0	0	0	0	0	0
Pt. 6	1	1	0	1	1	0	1	0	0	1	1	1	1	0	0	0	0	0
Pt. 7	1	1	0	1	1	0	0	0	0	0	0	0	0	0	0	0	0	0
Pt. 8	0	0	0	0	0	0	0	0	0	0	0	0	0	0	0	0	0	0
Pt. 9	0	0	0	0	0	0	0	0	0	0	0	0	0	0	0	0	0	0
Pt. 10	0	0	0	0	0	0	0	0	0	0	0	0	0	0	0	0	0	0
Pt. 11	1	0		2	1		0	0		0	0		0	0		0	0	
Pt. 12	0	0		1	0		0	0		0	0		0	0		0	0	
Pt. 13	1	0		0	0		0	0		0	0		0	0		0	0	
Pt. 14	0	0		0	0		0	0		1	1		0	0		0	0	
Pt. 15	1	0		0	0		0	0		0	0		0	0		0	0	

Adverse events (AEs) observed after FUS2 are reported for each patient at 1, 6, and 12 months follow-up (FU). Numbers within each cell indicate AE severity according to the Common Terminology Criteria for Adverse Events (CTCAE), version 5.0 (grade 0 = no AE; higher grades indicate increasing severity). Blank cells indicate follow-up time points not yet reached at the time of data analysis.

A subgroup analysis compared patients with gait disturbances persisting at 1 month (8/15, 53.3%) with those without gait disturbances (7/15, 46.7%). In T2 early imaging, median lesion volume after FUS2 was 82.8 mm3 (Q1–Q3: 56.9–107.8) in patients with persisting gait disturbances and 72.4 mm^3^ (Q1–Q3: 53.9–96.1) in those without gait disturbances (*p* = 0.95). In T2 late imaging, median lesion volume was 87.1 mm^3^ (Q1–Q3: 56.6–110.5) in patients with persisting gait disturbances and 80.8 mm^3^ (Q1–Q3: 58.9–101.9) in those without gait disturbances (*p* = 0.86). Similarly, the Euclidean distance between final FUS1 and FUS2 coordinates was comparable (*p* = 0.52) in patients with (median 0.8 mm, Q1–Q3: 0.7–1.6) and in those without gait disturbances persisting at 1 month (median 0.7 mm, Q1–Q3: 0.5–1.6). At baseline, axial SARA scores (gait and stance items) were comparable between the two groups. Over follow-up, both groups exhibited a transient worsening of axial SARA scores—higher in patients with persisting gait disturbance, as expected—with near-complete resolution at 12-month follow-up (Supplementary Table S4). Additional exploratory between-group analyses of clinical, demographic, and other procedural variables suggested significantly higher baseline tremor severity in patients with gait disturbance persisting at 1 month after FUS2, with no significant differences for the other variables (Table 3).

**Table 3:** Exploratory univariate analysis of demographic, clinical and procedural variables in patients with vs. those without gait disturbance persisting at 1-month follow-up (FU) after FUS2.

Variables	Patients without gait disturbance at 1-month FU (*n* = 7)	Patients with gait disturbance at 1-month FU (*n* = 8)	*P* value	Effect size
Demographic and clinical data				
Age at FUS2 (years)	71 [66–76]	77.5 [73.8–80.5]	0.12	0.42
Disease duration (years)	15 [11–32.5]	35 [27.5–50]	0.13	0.41
FUS1–FUS2 interval (months)	13 [12–15]	42 [5.8–61.3]	0.12	0.42
CRST total score pre FUS1	60 [53.5–65]	68 [67.0–74.5]	0.05	0.55
CRST total score pre FUS2	29.00 [25.5–32.5]	40.5 [38.8–47]	**<0** **.** **01**	0.79
Gait + stance (SARA items) pre FUS2^a^	0.00 [0–0.5]	0.50 [0–1]	0.39	0.24
Parameters of sonications with T mean ≥ 55 °C				
Mean Energy (J)	14,300 [12,183–1,875]	10,400 [8,800–14,525]	0.25	0.31
Mean Power (W)	800 [750–840]	685 [645–777.5]	0.15	0.39
Mean T ( °C)	54 [53.5–57]	55.3 [54.5–56]	1.00	0.02
Max T ( °C)	58[54.5–61.8]	60.00 [58.6–60]	0.56	0.17

Values are reported as median [interquartile range, Q1–Q3]. Between-group comparisons were performed using the Wilcoxon rank-sum test. Effect sizes were estimated using Wilcoxon rank-biserial correlation. Analyses were exploratory and not prespecified as efficacy outcomes.

CRST: Clinical Rating Scale for Tremor; J: Joule; n: number of patients; SARA: Scale for the Assessment and Rating of Ataxia; T: Temperature; W: Watt.

^a^ Sum score of SARA item 1 and 2.

Statistically significant *p*-values are shown in bold.

**Figure 3:**
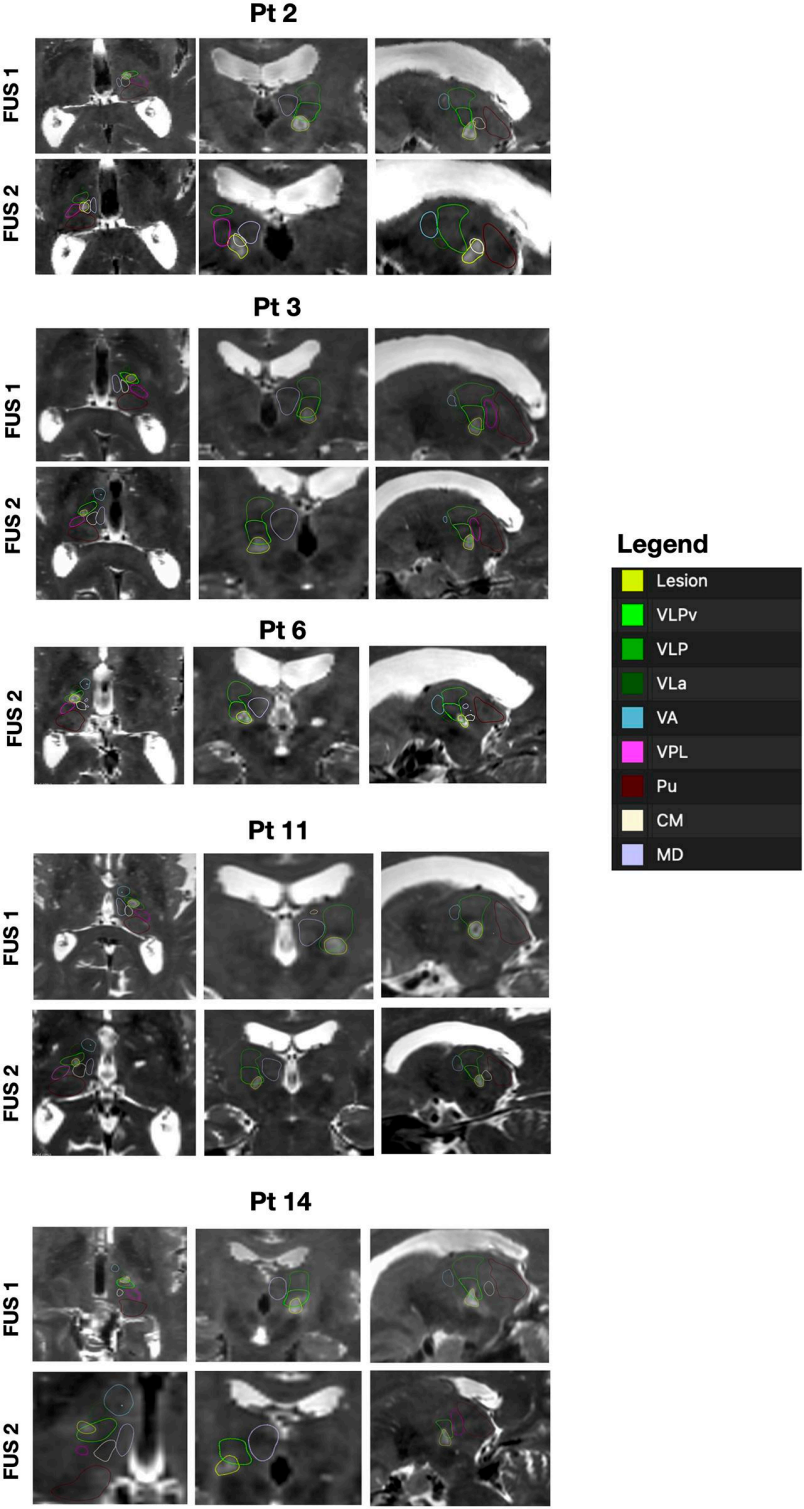
MRI lesion–nuclei relationships in patients with persistent adverse events. Representative axial, coronal, and sagittal MRI sections illustrating lesion location and spatial relationships with thalamic nuclei in five selected patients presenting with adverse events persisting at the last available follow-up (6 or 12 months). For each patient, images from both the first-side (FUS1) and second-side (FUS2) procedures are shown, with one representative slice per orthogonal plane extracted from late (1-month) post-treatment T2-weighted MRI. Images were selected using 3D Slicer by centring the cursor on the lesion core and adjusting each plane to optimise visualisation of the lesion and the most relevant adjacent anatomical structures. The ventral lateral posterior nucleus (VLP) is segmented in green, with the ventral subregion (VLPv) corresponding to the Vim outlined in bright green. Treatment-related lesions are segmented in yellow. Relevant neighbouring anatomical structures are indicated in the legend accompanying each patient panel. VLPv, ventral part of the ventral lateral posterior nucleus; VLP, ventral lateral posterior nucleus; VLa, ventral lateral anterior nucleus; VA, ventral anterior nucleus; VPL, ventral posterolateral nucleus; Pu, pulvinar; CM, centromedian nucleus; MD, mediodorsal nucleus.

Finally, a descriptive single-patient analysis was performed in patients presenting with persistent AEs at the last available follow-up. Individual clinical characteristics, targeting coordinates, inter-side displacement metrics, and lesion volumes after both FUS1 and FUS2 are reported in Table 4. With respect to lesion size, only one patient (patient 2) exhibited lesion volumes at 1-month follow-up exceeding the overall cohort mean after both FUS1 and FUS2, with values at the upper end of the cohort distribution, approaching two standard deviations above the group mean. In the remaining patients, lesion volumes fell within the overall cohort distribution at both early and late imaging time points. Qualitative inspection of lesion location on post-treatment MRI—based on T2 late—revealed heterogeneous spatial configurations across patients as represented in Figure 3. In patient 2, lesions after both FUS1 and FUS2 extended inferiorly beyond the intended Vim target, whereas in patient 11, inferior extension beyond the target was primarily observed after FUS2. In patient 3, the FUS2 lesion showed a posterior displacement relative to FUS1, approaching posterior sensory thalamic territories. Patients 6 and 14, both presenting with persistent gustatory disturbances, exhibited different spatial patterns: in patient 6, only FUS2 imaging was available, with the lesion extending medially toward sensory thalamic regions, whereas in patient 14 both FUS1 and FUS2 lesions appeared without clear involvement of sensory thalamic nuclei but more symmetric than others.

**Table 4:** Individual clinical and imaging characteristics of patients with persistent adverse events (AEs).

Pts	AEs (FU length)	Age (y)		Interval (m) between FUS1-FUS2	Final Coordinates^a^		Inter-side displacement^b^ (FUS2–FUS1)	Volume Lesion^c^ (mm^3^)		SDR	
		FUS1	FUS2		after FUS1	after FUS2		after FUS1	after FUS2	FUS1	FUS2
Pt 2	Dysarthria (12)	71	73	25	x: 15.0	x: 15.5	Δx: 0.5	24 h: 89.9	24 h: 111.4	0.69	0.68
					y: 9.1	y: 9.5	Δy: 0.4	1 m: 154.0	1 m: 166.2		
					z: 1	z: 1.5	Δz: 0.5				
							d: 0.81				
Pt 3	Dysesthesia (12)	69	70	17	x: 14.2	x: 14.0	Δx: −0.2	24 h: 58.0	24 h: 63.6	0.47	0.48
					y: 7.8	y: 8.3	Δy: 0.5	1 m: 75.0	1 m: 89.3		
					z: 1.5	z: 2	Δz: 0.5				
							d: 0.7				
Pt 6	Dysgeusia (12)	71	76	69	x: –	x: 16.0	Δx: –	24 h: –	24 h: 85.5	–	0.55
					y: –	y: 8.5	Δy: –	1 m: –	1 m: 85.0		
					z: –	z: 2	Δz: –				
							d: –				
Pt 11	Dysarthria (6)	84	89	59	x: 15	x: 14.5	Δx: −0.5	24 h: 81.6	24 h: 33.2	0.66	0.68
					y: 6.5	y: 8.6	Δy: 2.1	1 m: 114.2	1 m: 59.4		
					z: 1.5	z: 2.0	Δz: 0.5				
							d: 2.2				
Pt 14	Dysgeusia (6)	71	72	12	x: 13.5	x: 13.5	Δx: 0	24 h: 96.8	24 h: 64.1	0.46	0.48
					y: 8.5	y: 8.5	Δy: 0	1 m: 71.5	1 m: 80.8		
					z: 2	z: 2.5	Δz: 0.5				
							d: 0.5				

This table reports individual clinical, procedural, and imaging features of patients presenting with persistent AEs at the last available follow-up after FUS2. For patient 6, FUS1 imaging data were unavailable because the first-side procedure was performed at another centre.

ACPC, anterior commissure-posterior commissure; FU, follow-up; h, hours; m, months; mm, millimetres; y, years; Pt, patient.

^a^ *Stereotactic coordinates* are expressed as follows: x, distance (mm) from the AC–PC line; y, distance (mm) from the posterior commissure; z, distance (mm) orthogonal to the ACPC plane.

^b^ *Inter-side spatial displacement* between FUS1 and FUS2 is expressed as directional coordinate differences (Δx, Δy, Δz; FUS2–FUS1, in millimetres) and as the corresponding Euclidean distance (d, in millimetres), providing a scalar measure of overall targeting asymmetry.

^c^ *Volume lesion values* derived from lesion segmentation represent the mean of measurements obtained independently by the two raters.

### Literature review

Of the 169 records identified through the literature search, 10 studies reporting relevant outcomes were ultimately included in the qualitative synthesis and are summarised in Table 5, with a focus on targeting strategies for bilateral staged MRgFUS thalamotomy.

**Table 5:** Features of the studies included in the systematic review.

Study	Design	Follow-up (months)	Sample size	Minimum interval (months)	Mean interval (months)	Mean age (years)	Targeting and procedural strategy	Main adverse events (AEs)
Bruno et al. (32)	Case report	6	*n* = 1	24	24	63	Initial asymmetric targeting, followed by a final contralateral lesion explicitly mirrored to the first-side target.	No AEs reported
Martínez-Fernández et al. (23)	Prospective, open-label, multicentre study	6	*n* = 9	5	24 ± 18	71 ± 6	Symmetric targeting strategy, with comparable stereotactic coordinates and procedural steps for both hemispheres.	Gait: 4 (0) mild; 2^d^ (0) requiring walking assistance
								Dysarthria: 1^d^ (0)
								Dysphagia: none
								Sensory disturbances: 2 (2)
								Dysgeusia: 1 (0)
								Muscle weakness: none
Fukutome et al. (25)	Retrospective case series	3	*n* = 5	12	27.8 ± 11.5^c^	57.6 ± 17.5^c^	Asymmetric second-side targeting, with FUS2 planned slightly anterior and superior relative to FUS1 target.	Gait: none
								Dysarthria: 1 (0)
								Dysphagia: none
								Sensory disturbances: 2 (1)
								Dysgeusia: none.
								Muscle weakness: 1 (0)
Iorio-Morin et al. (4)	Prospective, single-arm, single-blinded, multicentre, phase 2 trial	3	*n* = 10	6	median 9, range 7–56	71.2 ± 7.5^c^	Asymmetric approach, with FUS 2 target intentionally positioned more dorsally (approximately 1 mm) and treated with a smaller planned lesion volume (i.e., a 5–6 mm spot diameter defined at the 51 °C isotherm on MR thermography).	Data included in the cohort by Sarica et al. (24)
Pearce et al. (33)	Case report	3, 6	*n* = 1	7	7	69	Asymmetric targeting strategy with a deliberate superior adjustment (+1 mm) for FUS2 compared with FUS1.	Gait: 1 (0) mild
								Dysarthria: 1 (0) mild
								Dysphagia: 1 (0) mild
								Sensory disturbances: none
								Dysgeusia: none
								Muscle weakness: none
Scantlebury et al. (30)	Prospective, open-label, single-centre study	median 4.4, range 3–6	*n* = 11	12	35.3 ± 23.6^c^	69.6 ± 8.6^c^	Not reported.	Gait: 4 (0) mild to moderate
								Dysarthria: none
								Dysphagia: 1 (0)
								Sensory disturbances: 4 (4) mild
								Dysgeusia: 1 (0) mild; 3 (3) moderate
								Muscle weakness: none
Kaplitt et al. (5)	Prospective, open-label, multicentre study	1, 3, 6, 12	*n* = 51	9	26.4 ± 19.2^b^	73 ± 9.3	Standardised targeting framework based on predefined stereotactic coordinate ranges, with FUS2 size and final position determined intraoperatively according to tremor response and early adverse effects rather than explicit symmetric or asymmetric planning.	Gait: 16 (7) mild; 1 (0) moderate
								Dysarthria: 14 (7) mild; 1 (0) moderate
								Dysphagia: 3 (2) mild; 1 (1) moderate
								Sensory disturbances: 18 (9) mild
								Dysgeusia: 10 (5) mild; 1 (1) moderate
								Muscle weakness: 2 (0) mild
^a^Sarica et al. (24)	Retrospective-prospective case series	23.4 ± 13.3	*n* = 19	6	17.3 ± 15.7	69.8 ± 10.3	Asymmetric approach, with FUS 2 target intentionally positioned more dorsally (approximately 1 mm) and treated with a smaller planned lesion volume (i.e., a 4–6 mm spot diameter defined at the 51 °C isotherm on MR thermography).	Gait: 4 (3) grade 1
								Dysarthria: 2 (2) grade 1
								Dysphagia: 5 (3) grade 1
								Sensory disturbances: 2 (1) grade 1
								Dysgeusia: 4 (3) grade 1; 1 (1) grade 2
								Muscle weakness: 1 (0) grade 2
Campins-Romeu et al. (6)	Prospective, open-label, single-centre study	6	*n* = 20	9	17.8 ± 7.6	66.5 ± 9.2	Asymmetric FUS targeting with a systematic rostral and superior adjustment (+1 mm) relative to the FUS1.	Gait: 3 (3) mild
								Dysarthria: 2 (1) mild
								Dysphagia: none
								Sensory disturbances: 5 (5) mild
								Dysgeusia: none
								Muscle weakness: 1 (0) mild
Pertsch et al. (26)	Retrospective case series	6, 12, 24+	*n* = 30	6	Median 11.6 range 6–21.6	71.2 ± 8.2	Symmetric initial stereotactic targeting coordinates, combined with a less aggressive FUS2 treatment strategy characterised by lower total delivered energy and frequent avoidance of tandem lesioning.	Gait: 12 (4) grade 1; 1 (1) grade 2
								Dysarthria: 21 (4) grade 1; 3 (1) grade 2
								Dysphagia: 9 (1) grade 1; 1 (0) grade 2
								Sensory disturbances: 15 (2) grade 1
								Dysgeusia: 7 (0) grade 1; 1 (1) grade 2
								Muscle weakness: none

Data are reported as mean ± SD unless otherwise stated. AEs are reported as described in the original studies as AEs (persistent AEs), at last available follow-up, with severity grading as reported by Authors.

^a^ Includes data from Iorio-Morin et al., prospective phase 2 trial (NCT04501484) (4).

^b^ Interval originally reported in years and converted to months.

^c^ Calculated from the original dataset.

^d^ Worsening of pre-existing symptoms.

mm: millimetres; n: number of patients; SD: standard deviation.

## Discussion

In this retrospective-prospective single-centre study of patients with ET undergoing staged bilateral MRgFUS thalamotomy, we provide a direct comparison between FUS1 and FUS2 comparison regarding procedural, stereotactic, and MRI-based lesion features. Three main findings emerged. First, FUS2 was performed under largely comparable anatomical and technical conditions, with no significant differences in lesion volumes at early and late T2-weighted imaging. Second, starting from mirrored stereotactic coordinates, final FUS2 targeting showed a small but consistent anterior–dorsal shift, reflecting clinically driven refinement rather than strict symmetric replication. Third, AEs after FUS2 were predominantly mild and transient, with persistent gait disturbance at 1 month not being associated with clear volumetric differences or inter-side targeting-displacement.

Although the present study was not designed to assess clinical efficacy, the imaging and procedural findings were paralleled by clinically meaningful tremor control, with substantial improvement in the non-dominant hand tremor and additional benefit on head and voice tremor after FUS2, as better detailed in a separate manuscript focused on clinical outcomes (7).

### Procedural aspects

Procedure duration and the number of sonications tended to be lower during FUS2, possibly reflecting increased procedural experience related to the team's learning curve and the availability of FUS1 data. The availability of mirrored stereotactic coordinates and prior lesion imaging provides a high-confidence anatomical reference, reducing the need for extensive exploratory sonications to localise the effective target during FUS2. In our cohort, key imaging-derived anatomical parameters relevant to acoustic transmission (e.g., SDR) were comparable between FUS1 and FUS2. Consistently, sonication characteristics associated with effective lesioning (i.e., delivered energy, acoustic power, and achieved temperatures) largely overlapped across sides. Notably, in a subset of patients, target temperatures of 55 °C could not be reached during FUS2 despite favourable clinical and radiological outcomes, suggesting the effectiveness of the treatment even in the absence of maximal thermal sonications.

### Lesion volume and reliability of MRI-based quantification

From a post-interventional imaging perspective, lesion morphology and volume were highly consistent between FUS1 and FUS2. Lesion volumes measured in T2 early and T2 late imaging did not differ significantly between sides and showed a comparable temporal evolution characterised by progressive volume reduction. Using a standardised, atlas-based thalamic segmentation approach, MRI-based volumetric assessment demonstrated excellent inter-rater agreement (21) across procedures and time points, with agreement further improving in T2 late imaging. The slightly greater variability observed on early MRI is biologically and methodologically plausible, reflecting transient peri-lesional changes in the acute post-procedural phase, whereas lesion geometry appeared more stable at later imaging.

### Targeting strategies

From a targeting perspective, staged bilateral lesioning may benefit from a safety-oriented approach that does not rely on strict spatial symmetry between the two lesions, in line with long-standing principles of functional lesioning surgery cautioning against indiscriminate bilateral intervention on homologous structures (22). In our cohort, the second-side procedure was therefore initiated using mirrored stereotactic coordinates as a reproducible starting reference but was typically concluded with a deliberately planned small dorsal adjustment, and in some cases, an anterior refinement guided by intraoperative clinical response, reflecting incremental procedural adjustments over time. This approach is consistent with the literature on staged bilateral MRgFUS thalamotomy for ET, as documented by the results of the systematic review. Indeed, across published series, second-side MRgFUS thalamotomy was rarely performed as a purely symmetric replication of the first procedure (23). Most groups have adopted some degree of spatial or procedural asymmetry aimed at enhancing safety, ranging from modest dorsal (4, 6, 24) and/or anterior (25) target adjustments to the use of symmetric coordinates combined with more conservative procedural parameters, such as reduced delivered energy (26) or stricter thermal stopping rules (4, 24). Collectively, these strategies reflect a shared effort to balance safety and efficacy in the bilateral setting. Within this framework, the small but consistent anterior–dorsal shift observed in our cohort should be interpreted as a clinically informed refinement of FUS2 targeting rather than a deviation from established Vim-based stereotactic paradigms.

### Imaging interpretation of AEs

Consistent with previously published staged bilateral MRgFUS series, AEs after FUS2 were predominantly mild and transient, with gait disturbance representing the most frequent early post-procedural symptom. In exploratory subgroup analyses, neither lesion volume at T2 early and T2 late, nor inter-side targeting displacement quantified using final stereotactic coordinates, differed significantly between patients with and without gait disturbance persisting at the 1-month follow-up. These findings suggest that simple volumetric or inter-side spatial symmetry metrics may be insufficient to explain gait disturbances, which are more frequently reported after bilateral than unilateral procedures (i.e., approximately 28.5% according to our experienc e^11^). Notably, in our cohort, gait disturbances were fully reversible over time, supporting the hypothesis that such symptoms may reflect transient functional effects, including delayed resolution of vasogenic oedema, rather than permanent structural injury. These effects may be further modulated by inter-individual differences in axial functional reserve. In this context, although our exploratory univariate analysis suggests a potential association between greater baseline tremor severity and the development of gait disturbances, consistent with observations reported after unilateral thalamotomy (27), these findings remain speculative and hypothesis-generating, as they do not imply an independent or causal relationship. Larger datasets and integrative analyses incorporating multiple variables (e.g., age, disease duration, inter-procedural interval, lesion volume and location) will be required to better delineate individual risk profiles.

In addition, the exploratory qualitative assessment at the single-patient level highlighted the relevance of lesion location and spatial configuration when interpreting AEs after bilateral procedures. Specifically, persistent AEs may reflect lesion extension into adjacent structures [e.g., posterior subthalamic area or sensory thalamic territories (28)], while broader network-level effects related to bilateral thalamic intervention may also contribute in selected cases. In this regard, historically, dysarthria has been reported more frequently after bilateral thalamic surgeries (29). Similarly, gustatory disturbances (dysgeusia) have also been described in both bilateral MRgFUS and Deep Brain Stimulation (30, 31), exemplifying the overlap between spatial and network-level mechanisms. These mechanisms are not mutually exclusive, and their relative contribution likely varies across patients, underscoring the need for cautious attribution of AEs after FUS2.

### Strengths and limitations

This study provides a comprehensive radiological characterisation of staged bilateral MRgFUS thalamotomy using a standardised, atlas-based thalamic segmentation approach. Lesion delineation was performed through a semi-automated pipeline with excellent inter-rater agreement, supporting the robustness and reproducibility of MRI-based volumetric measurements. The within-patient design allowed direct comparison between FUS1 and FUS2, minimising inter-individual anatomical variability. Several limitations should be acknowledged. Clinical and imaging data related to FUS1 were retrieved retrospectively, and baseline information was unavailable for a small subset of patients. Lesion segmentation relied on semi-automated methods with manual refinement; although blinded double rating mitigated operator dependence, intra-rater reliability was not formally assessed. Inter-side spatial analyses were based on final stereotactic targeting coordinates rather than true lesion centroids, potentially underrepresenting post-procedural lesion geometry. In addition, tractography data were unavailable, as diffusion imaging was not part of the routine protocol, and minor variability in T2 early timing may have influenced acute lesion appearance. In addition, the relatively small sample size and incomplete availability of 12-month follow-up data limited statistical power and hindered meaningful correlation analyses between lesion characteristics and clinical outcomes. Accordingly, the present study was neither designed nor powered to support multivariable correlation or predictive analyses. Gait and balance were not formally assessed using instrumental analyses, while clinical efficacy outcomes (i.e., tremor severity) were not analysed, as the study was specifically designed to address procedural and radiological aspects of staged bilateral procedure.

## Conclusion

In this single-centre cohort, staged bilateral MRgFUS thalamotomy for essential tremor was associated with comparable stereotactic targeting conditions, sonication parameters, and lesion volumes between FUS1 and FUS2. Atlas-based volumetric and spatial analyses demonstrated high reproducibility of lesion measurements and did not show systematic differences in lesion size or gross inter-side targeting consistency. Persistent AEs were uncommon and might have been influenced by lesion geometry and spatial extension, in addition to potential network-level effects of bilateral lesion. Collectively, these findings support the feasibility of staged bilateral MRgFUS when performed using a cautious, imaging-guided targeting strategy that allows clinically informed refinements of second-side targeting. They also highlight the value of detailed post-procedural MRI analysis in disentangling bilateral effects from target placement- and lesion-shape-related mechanisms, and in guiding future efforts to optimise safety in bilateral MRgFUS thalamotomy.

## Data Availability

The original contributions presented in the study are included in the article/Supplementary Material, further inquiries can be directed to the corresponding author.
