# Supplementary material for: Imaging and procedural features of staged bilateral magnetic resonance-guided focused ultrasound thalamotomy for essential tremor
Source: Front Radiol. 2026 Mar 27;6:1779005. doi: 10.3389/fradi.2026.1779005 (PMC13066307; doi:10.3389/fradi.2026.1779005)
Supplement: Supplementary file 1 [file Datasheet1.pdf]

Imaging and Procedural Features of Staged Bilateral Magnetic Resonance-guided Focused Ultrasound Thalamotomy for Essential Tremor

Fabio Paio*[1,2], Antonio Ambrosin[3], Giorgia Bulgarelli[4], Micaela Tagliamonte[3], Chiara Zucchella[2], Elisa Mantovani[1,2], Tommaso Bovi[2], Michele Longhi[4], Antonio Nicolato[4], Emanuele Zivelonghi[5], Luisa Altabella[5], Carlo Cavedon[5], Francesco Sala[6,7], Benedetto Petralia[3], Bruno Bonetti[2], Michele Tinazzi[1,2], Stefano Tamburin†[1,2], Giuseppe K Ricciardi†[3]

[1] Neurology Section, Department of Neurosciences, Biomedicine, and Movement Sciences, University of Verona, Verona, Italy
[2] Neurology Unit, Department of Neurosciences, Azienda Ospedaliera Universitaria Integrata, Verona, Italy
[3] Neuroradiology Unit, Department of Pathology and Diagnostics, Azienda Ospedaliera Universitaria Integrata, Verona, Italy
[4] Stereotactic Neurosurgery and Radiosurgery Unit, Department of Neurosciences, Azienda Ospedaliera Universitaria Integrata, Verona, Italy
[5] Medical Physics Unit, Department of Pathology and Diagnostics, Azienda Ospedaliera Universitaria Integrata, Verona, Italy
[6] Neurosurgery Section, Department of Neurosciences, Biomedicine, and Movement Sciences, University of Verona, Verona, Italy
[7] Neurosurgery Unit, Department of Neurosciences, Azienda Ospedaliera Universitaria Integrata, Verona, Italy

†Shared Senior Authorship

*Corresponding author
Fabio Paio, MD, FEBN
Department of Neurosciences, Biomedicine and Movement Science,
University of Verona
Piazzale Ludovico Antonio Scuro, 10 – 37124, Verona, Italy
E-mail: fabio.paio@univr.it

Supplementary Method: MRI detailed protocols

3D T1-weighted (BRAVO)

- Field strength: 3.0 T
- Sequence type: 3D T1-weighted
- TR: 11.44 ms (BRAVO)
- TE: 4.7 ms
- TI: 450 ms
- Flip angle: 12°
- Voxel size: 1.0 × 1.0 × 1.5 mm
- Slice thickness: 1.5 mm
- Matrix: 288 × 288
- FOV: 240mm
- Acquisition time: 4 minutes 28 seconds
- Parallel imaging: yes (ARC 2)

3D T2-weighted (CUBE)

- Sequence type: 3D T2-weighted
- TR: 3002 ms
- TE: 97.44 ms
- Echo train length: 130
- Voxel size: 0.8 × 0.8 × 1.4 mm
- Slice thickness: 1.4 mm
- Matrix: 288 x 288
- FOV: 240 mm
- Acquisition time: 3 minutes 25 seconds
- Parallel imaging: yes (ARC 2)
- Fat suppression: yes

Supplementary Table S1: Common Terminology Criteria for Adverse Events (CTCAEv5) definitions and grading

CTCAE Term	Grade 1	Grade 2	Grade 3	Grade 4	Grade 5
**Gait disturbance**<br>Definition: A disorder characterized by walking difficulties.	Mild change in gait (e.g., wide-based, limping or hobbling)	Moderate change in gait (e.g., wide-based, limping or hobbling); assistive device indicated; limiting instrumental ADL	Limiting self-care ADL	-	-
**Dysarthria**<br>*A disorder characterized by slow and slurred speech resulting from an inability to coordinate the muscles used in speech.*	Mild slurred speech	Moderate impairment of articulation or slurred speech	Severe impairment of articulation or slurred speech	-	-
**Dysphagia**<br>*A disorder characterized by difficulty in swallowing.*	Symptomatic, able to eat regular diet	Symptomatic and altered eating/swallowing	Severely altered eating/swallowing; tube feeding, TPN, or hospitalization indicated	Life-threatening consequences; urgent intervention indicated	Death
**Dysesthesia**<br>*A disorder characterized by distortion of sensory perception, resulting in an abnormal and unpleasant sensation.*	Mild sensory alteration	Moderate sensory alteration; limiting instrumental ADL	Severe sensory alteration; limiting self-care ADL	-	-
**Dysgeusia**<br>*A disorder characterized by abnormal sensual experience with the taste of foodstuffs; it can be related to a decrease in the sense of smell.*	Altered taste but no change in diet	Altered taste with change in diet (e.g., oral supplements); noxious or unpleasant taste; loss of taste	-	-	-
**Muscle weakness**<br>*A disorder characterized by a reduction in the strength.*	Symptomatic; perceived by patient but not evident on physical exam	Symptomatic; evident on physical exam; limiting instrumental ADL	Limiting self-care ADL	-	-

Legend: ADL: Activities of Daily Living; TPN: Total Parenteral Nutrition

Supplementary table S2: Observed CRST and QUEST scores across different timepoints

		Pre-FUS1 (n = 14)	Pre-FUS2 (n = 15)	1 Month (n = 15)	6 Months (n = 15)	12 Months (n = 10)
**CRST total score**		65.3 ± 8.3	36.5 ± 9.0	14.4 ± 7.0	16.1 ± 7.4	15.8 ± 6.7
**Hand treated by FUS2***	CRST A	7.6 ± 2.0	8.0 ± 2.1	1.6 ± 1.1	2.2 ± 1.2	1.7 ± 0.8
	CRST B	12.5 ± 2.5	13.0 ± 2.4	5.5 ± 2.6	6.3 ± 3.3	6.6 ± 3.1
	CRST A + B	20.1 ± 3.9	21.0 ± 4.1	7.1 ± 3.4	8.5 ± 4.2	8.3 ± 3.7
**Head tremor**	CRST A	1.9 ± 0.8	1.7 ± 0.8	0.3 ± 0.5	0.3 ± 0.5	0.4 ± 0.5
**Voice tremor**	CRST A	1.6 ± 0.5	1.6 ± 0.5	0.9 ± 0.4	0.9 ± 0.3	0.9 ± 0.3
**Functional disability**	CRST C	18.4 ± 2.5	7.3 ± 2.7	1.3 ± 1.3	1.7 ± 1.6	2.0 ± 1.7
**QUEST**		57.1 ± 16.2	30.5 ± 13.5	9.5 ± 8.5	9.6 ± 8.7	10.8 ± 11.4

Legend. *All second-side procedures were performed on the right thalamus to treat left-hand tremor.

For each outcome, the *observed mean ± standard deviation (SD)* is reported; Pre-FUS1 = baseline data before first side thalamotomy; Pre-FUS2 = baseline data before second-side thalamotomy; 1, 6, 12 months = follow-up visits after the second procedure.

Abbreviations: CRST = Clinical Rating Scale for Tremor; n: number of patients; QUEST = Quality of Life in Essential Tremor Questionnaire.

Supplementary Table S3: Inter-rater reliability and agreement for lesion volume measurements

Comparison of Lesion Volume	No. of paired observations	ICC				Bland-Altman			
		Value	95% CI (lower)	95% CI (upper)	P-value	mean bias (rater 1 – rater 2)	Lower LoA	Upper LoA	SD differences
Within 24 hours after FUS1	13	0.91	0.75	0.97	p < 0.001	1.04	-16.12	18.21	8.76
At 1 month after FUS1	13	0.99	0.99	1.00	p < 0.001	1.24	-5.91	8.39	3.65
Within 24 hours after FUS2	15	0.95	0.84	0.98	p < 0.001	5.2	-14.26	24.66	9.93
At 1 month after FUS2	15	0.99	0.95	1.00	p < 0.001	2.7	-2.49	8.04	2.69

Legend. Inter-rater agreement for lesion volume measurements obtained independently by two blinded raters was assessed using the intraclass correlation coefficient (ICC) and Bland–Altman analysis. ICC values are reported with 95% confidence intervals. Bland–Altman results include mean bias (rater 1 – rater 2) and limits of agreement (LoA).

Abbreviations: ICC[2,1]: intraclass correlation coefficient; CI: confidence interval; LoA: limits of agreement; SD: standard deviation.

Supplementary Table S4: Longitudinal trends of axial SARA items in patients with and without gait disturbance persisting at 1 month after FUS2

	Patients without gait disturbance at 1-month (n = 7)	Patients with persisting gait disturbance at 1-month (n = 8)	P value
Gait (SARA item 1)			
Pre-FUS2	0 [0-1]	0.5 [0-1]	0.16
After 1 month	1 [0-1]	1 [1-1.5]	0.43
After 6 months	0 [0-1]	1 [0-1]	0.07
After 12 months*	0 [0-0]	0 [0-1]	0.22
Stance (SARA item 2)			
Pre-FUS2	0 [0-0]	0 [0-1]	0.92
After 1 month	0 [0-1]	1 [0.5-1]	0.22
After 6 months	0 [0-0]	0.5 [0-1]	0.16
After 12 months*	0 [0-1]	1 [1-1]	0.51
Gait + Stance (sum of SARA items 1 and 2)			
Pre-FUS2	0 [0-1]	0.5 [0-1]	0.39
After 1 month	1 [0-2]	2 [1.5-2.5]	0.28
After 6 months	0 [0-1]	1 [1-1.5]	**0.02**
After 12 months*	0 [0-0]	0 [0-1]	0.17

Legend: Patients were stratified according to the presence or absence of gait disturbance persisting at 1-month follow-up after FUS2. Axial function was explored using selected axial items of the Scale for the Assessment and Rating of Ataxia (SARA): gait (item 1), stance (item 2), and their sum (items 1 + 2). All patients had normal sitting ability (item 3 = 0). Values are reported as median [interquartile range, Q1–Q3]. Between-group comparisons were performed using the Wilcoxon–Mann–Whitney test. P values are reported for exploratory purposes only, and no correction for multiple comparisons was applied.

* A total of 10 patients (i.e., 5 in each group) reached the 12-month follow-up.
